# Insights into the N-Sulfation Mechanism: Molecular Dynamics Simulations of the N-Sulfotransferase Domain of Ndst1 and Mutants

**DOI:** 10.1371/journal.pone.0070880

**Published:** 2013-08-05

**Authors:** Tarsis F. Gesteira, Laércio Pol-Fachin, Vivien Jane Coulson-Thomas, Marcelo A. Lima, Hugo Verli, Helena B. Nader

**Affiliations:** 1 Departamento de Bioquímica, Universidade Federal de São Paulo, São Paulo, Brazil; 2 Centro de Biotecnologia, Universidade Federal do Rio Grande do Sul, Porto Alegre, Brazil; University of Cantebury, New Zealand

## Abstract

Sulfation patterns along glycosaminoglycan (GAG) chains dictate their functional role. The *N*-deacetylase *N*-sulfotransferase family (NDST) catalyzes the initial downstream modification of heparan sulfate and heparin chains by removing acetyl groups from subsets of *N*-acetylglucosamine units and, subsequently, sulfating the residual free amino groups. These enzymes transfer the sulfuryl group from 3′-phosphoadenosine-5′-phosphosulfate (PAPS), yielding sulfated sugar chains and 3′-phosphoadenosine-5′-phosphate (PAP). For the N-sulfotransferase domain of NDST1, Lys833 has been implicated to play a role in holding the substrate glycan moiety close to the PAPS cofactor. Additionally, Lys833 together with His716 interact with the sulfonate group, stabilizing the transition state. Such a role seems to be shared by Lys614 through donation of a proton to the bridging oxygen of the cofactor, thereby acting as a catalytic acid. However, the relevance of these boundary residues at the hydrophobic cleft is still unclear. Moreover, whether Lys833, His716 and Lys614 play a role in both glycan recognition and glycan sulfation remains elusive. In this study we evaluate the contribution of NDST mutants (Lys833, His716 and Lys614) to dynamical effects during sulfate transfer using comprehensive combined docking and essential dynamics. In addition, the binding location of the glycan moiety, PAPS and PAP within the active site of NDST1 throughout the sulfate transfer were determined by intermediate state analysis. Furthermore, NDST1 mutants unveiled Lys833 as vital for both the glycan binding and subsequent N-sulfotransferase activity of NDST1.

## Introduction

Sulfotransferases (STs) are a large family of enzymes that catalyze sulfate conjugation to carbohydrates, proteins, and a variety of metabolic compounds. Glycosaminoglycan STs transfer the sulfuryl group from the donor 3′-phosphoadenosine 5′-phosphosulfate (PAPS) to sugar chains, yielding 3′-phosphoadenosine 5′-phosphate (PAP) and sulfatede glycan. The high structural diversity of heparan sulfate (HS) implicates its functional roles in diverse biological events related to intracellular signaling, cell-cell interactions, tissue morphogenesis, binding to a variety of molecules, among others [1], [2]. Both sequence singularity, such as for binding to FGF or antithrombin, as well as by the spatial distribution of sulfate groups through the HS chains contribute to the diverse range of activity of HS [3], [4].

**Figure 1:**
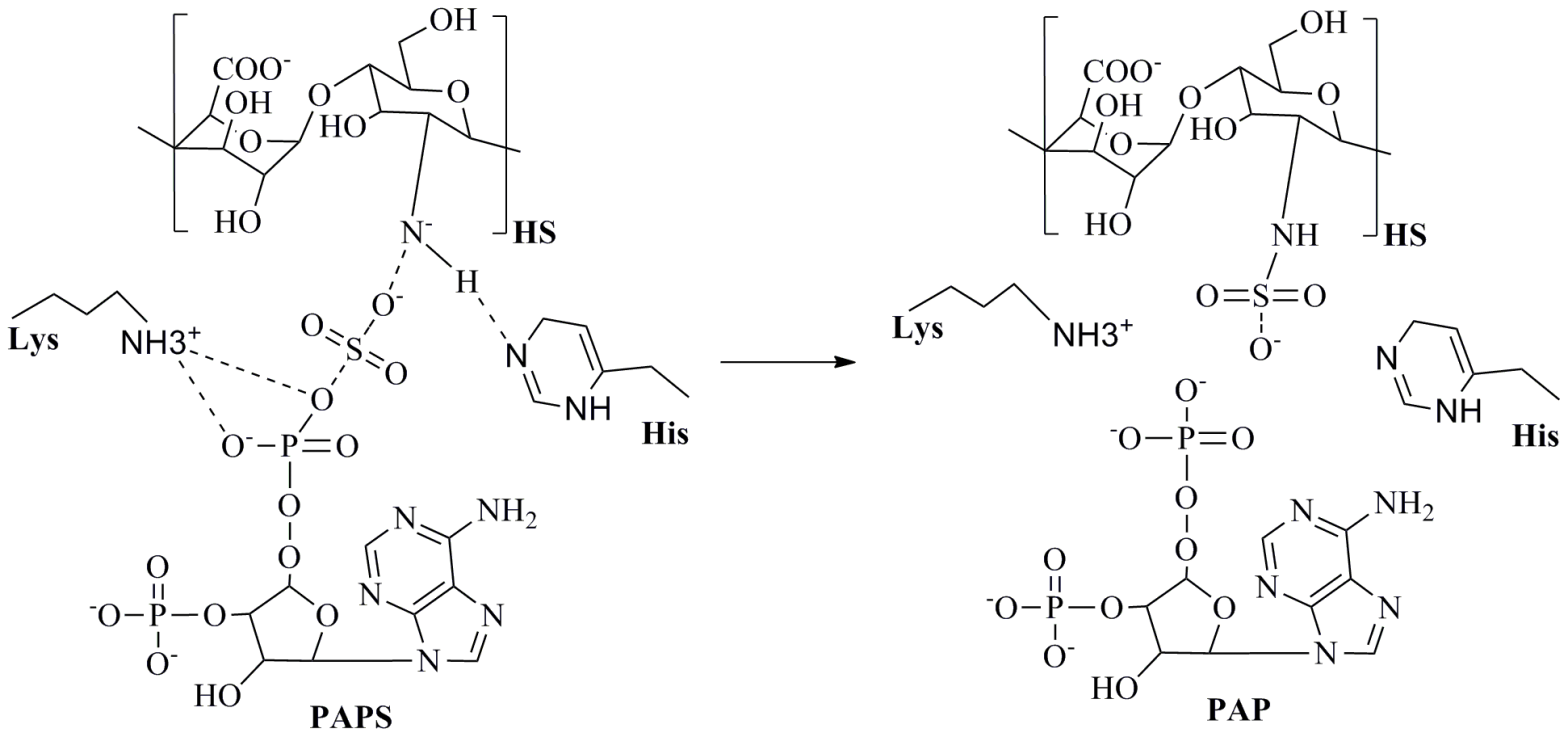
General reaction catalyzed by the NSTs.

The biosynthesis of HS and the related heparin starts in the Endoplasmatic Reticulum (ER) by the attachment of a β-D-xylosyl residue to the side chain oxygen atom of a serine residue in the core protein by xylosyltransferase [5], [6]. Then, galactosyltransferase I transfers the first galactose monosaccharide Galβ1,4 to the xylose residue, followed by the addition of a second galactose Galβ1,3 by a different enzyme, galactosyltransferase II. The linkage tetrasaccharide is terminated by the addition of a glucuronic acid residue by glucuronosyltransferase I. Thereafter, heparan sulfate chain polymerization starts with the addition of a *N*-acetylglucosamine (GlcNAc) and glucuronic acid (GlcA) residues by exostosin 1 and 2 (EXT1 and EXT2), followed by secondary modifications, including N-deacetylation and N-sulfation of GlcNAc, C5 epimerization of β-D-glucuronic acid to form α-L-iduronic acid(IdoA), 2-O-sulfation of IdoA or GlcA residues, and 6-O-sulfation and 3-O-sulfation of glucosamine residues. Sulfotransferases catalyze the transfer of a sulfuryl group from PAPS to substrates via an in-line ternary displacement reaction mechanism (Fig. 1), which is formed before the products are released. However, whether this occurs through an associative mechanism [bimolecular nucleophilic substitution (SN2)-like] or by a dissociative [unimolecular nucleophilic substitution (SN1)-like] mechanism [7]–[9] remains elusive. Once PAPS binds to the substrate, a conserved serine residue interacts with a conserved lysine residue, removing the nitrogen from the bridging oxygen side-chain and consequently preventing PAPS hydrolysis [10], [11]. Following the substrate binding, a conserved histidine deprotonates this acceptor, prompting the sulfur atom for the PAPS attack [9], [10], building a negative charge on the bridging oxygen atom from PAPS and so assisting its dissociation by interaction with the conserved serine [7], [9]. While it is still unknown whether this mechanism occurs in a sequential or random manner, recent reports have demonstrated the influence of many residues in this process, notably, two lysine residues stabilize the transition state by interacting with the bridging oxygen between the sulfate and phosphate groups of PAPS [12], [13].

The resolved tertiary complexes of both cytosolic and membrane-bound STs unveiled that they are single α/β globular proteins with a characteristic five-stranded parallel β-sheet [4], [14]. The β-sheet constitutes the PAPS-binding site and the core of the catalytic site, both of which are composed of conserved residues for both cytosolic and membrane-bound STs. However, the precise catalytic relevance of the boundary residues through the hydrophobic cleft is still unclear, as well as its significance to glycan recognition and sulfation.

In the present paper, the binding modes of different N-sulfotransferase mutants was investigated using molecular docking and essential dynamics aiming to define the binding site location of the glycan moiety, as well as determine the role of critical amino acid residues for ligand binding.

The glycosaminoglycan sulfation disposition and density is dictated by various factors, including: (i) availability/positioning of the acceptor (PAPS) within the enzyme active site; (ii) recognition/orientation of specific domains along the glycan chain within the enzyme active site; (iii) physical interaction of the enzyme with other enzymes involved in the GAG biosynthesis at the Golgi membrane. These concurrent events pose a challenge in determining the specific role of each player in the downstream modifications to the glycan chains, thereby, compelling the development of novel techniques, such as, applied theoretical methods which enables detailed analysis of isolated points in the process. Moreover, combining essential dynamics with molecular dynamics enables the study of conformational ensembles, as well as, deconvolution of the structural and the dynamic properties of the sulfate transfer reaction.

## Results

### Disaccharide Docking

**Figure 2:**
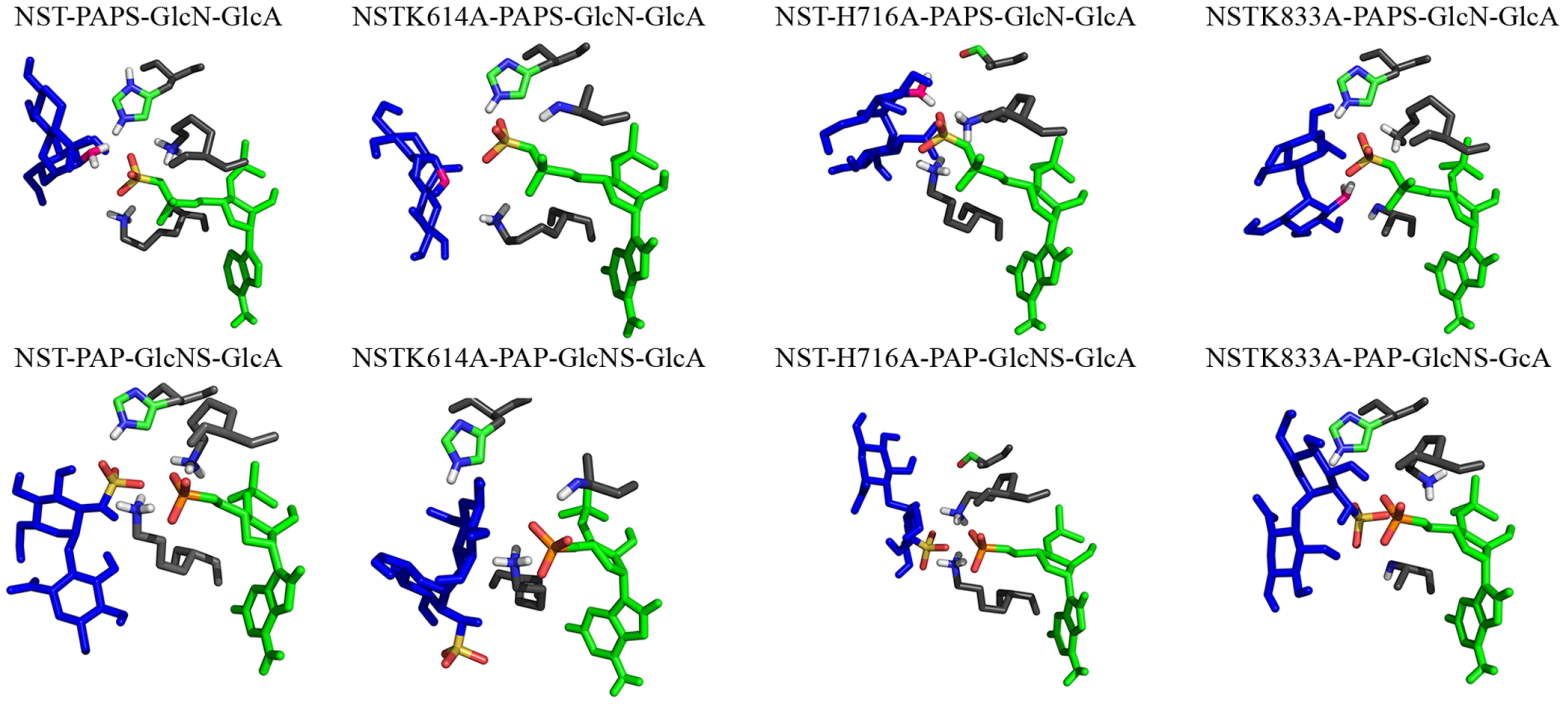
Interactions of N-sulfotransferase domain in NST1 bound to PAPS and PAP with the heparan disaccharide, as predicted by AutoDock. The disaccharide is shown as blue sticks, with sulfate as yellow and amide atoms as pink; PAPS and PAP are shown as green sticks with sulfate as yellow or phosphate as orange. Key reaction residues for enzyme function are shown as gray sticks.

Gorokhov and co-workers [13] have shown that the structural requirements for NST binding to GAGs includes mainly the residues in the 5′ phosphosulfate loop (5′-PSB loop) and the 3′ phosphate loop (3′-PB loop). Thus, for the docking experiments, the sulfuryl group was added to the PAP molecule before the disaccharide docking, resulting in a specular approach of catalytic residues to the substrate. The interaction modes of the α-GlcN-(1→4)-GlcA and NST are shown in Fig. 2, Fig. S1 and the distances listed in Table 1, where only the mutated amino acids are displayed. Two-dimensional plots of the catalytic domain displaying PAPS, PAP and disaccharide interacting amino acids and bridging water molecules with details of hydrogen bond distances were created using LIGPLOT [15] and displayed in Fig. S2a–c. The docking confirmed previous results of the involvement of Glu641, His716 and Arg835 on ligand binding site [13]. Also, it showed that both Lys614 and Lys833 formed a hydrogen bond with Oγ from PAPS. Moreover, the His716Ala mutant showed an increased length of this bond, to 2.1 Å. This increase in glycan/PAPS interaction was also evidenced for the other three docking mutants, as shown in Table 1. Based on the docking experiments with the Lys833Ala mutant, our results suggest that residues Lys614 and Lys833 are primarily responsible for both sulfate stabilization as well as glycan binding, implying its role potential role in neutralizing the sulfuryl group. Moreover, the His716 residue not only plays a role on glycan binding, but also as the basic residue required for stabilizing the binding site cleft.

**Table 1:** N-sulfotransferase 1 and mutants docking energies and hydrogen bond distances.

Enzyme/GAG System	Interacting atoms			Distance (Å)
	NST amino acids	α-GlcN-(1→4)-GlcA or α-GlcN-(1→4)-GlcA	PAPS or PAP	
NSTPAPSα-GlcN-(1→4)-GlcA		GlcN:NγH2a	PAPS:O1S	1.8
		GlcN:O6H6*	PAPS:O2′	2.1
		GlcN:O6B	PAPS:H2′	1.9
	Arg835:NHη22	GlcN:O2B		2.3
	His716: NHτ	GlcN:O4H4*		2.2
	Lys833: NHΖ3		PAPS:O5C	2.0
	Lys614: NHΖ3		PAPS:O5C	1.9
NST614APAPSα-GlcN-(1→4)-GlcA	His720: NHτ	GlcN:O6B		2.1
	His 716: NHτ	GlcN:O5		2.1
	Glu641:OE1	GlcA:O3H3		1.9
		GlcN:O1H1	PAPS O	2.1
	Ser832:OHγ	GlcN:O4		2.2
	Ser832:OHγ	GlcN:O4H4*		1.8
	Lys833: NHΖ3		PAPS:O5C	2.0
NST716APAPSα-GlcN-(1→4)-GlcA		GlcN:O2H2	PAPS:O	2.2
		GlcN: O3H3	PAPS:O	2.1
	Glu641:OE1	GlcN:O6H6*		1.7
		GlcN:O4H4*	PAPS:O	2.1
NST833APAPSα-GlcN-(1→4)-GlcA		GlcN:O6H6*	PAPS:O	1.9
	His716:NE2	GlcN:O4H4*		1.8
	His716:NE2	GlcA:O3H3*		2.3
NSTPAPα-GlcNS-(1→4)-GlcA	Glu641:OE1	GlcA:O4H4*		2.0
	Glu641:OE2	GlcN:O2H2		2.4
	Lys614:HZ2		PAP:O5C	2.0
NST614APAPα-GlcN-(1→4)-GlcA	Glu641:OE1	GlcA:O6H6*		2.1
	Ser832:OG	GlcN:O4H4*		1.9
	Glu641:OE2	GlcN:O2H2		2.2
NST716APAPα-GlcN-(1→4)-GlcA	Gln613:HE21	GlcN:O4H4*		
	Arg835:HH22	GlcA:O6A		1.8
	Lys614:HZ3		PAP:O5C	1.8
	Glu641:OE1	GlcA:H2		2.1
	His720:HE2	GlcA:O6A		2.2
	Ser832:HG	GlcA:O5/O1		1.8/1.7
	Glu614:OE1	GlcA:O3H3*		2.2
NST833APAPα-GlcN-(1→4)-GlcA	Glu641:OE1	GlcN:O6H6*		2.3
	Glu641:OE1	GlcN:O4H4*		2.2
	Cys828:O	GlcA:O1H1		2.2

* see Fig. S7 for atom labels.

The docking calculations for the PAP/*α-*GlcNS-(1→4)-GlcA system clearly indicate that the same hydrogen bonds and molecular orientations are present in both PAPS and PAP binding. Comparing the docking energies of NST to each NST mutant, we found that the His716 residue mutation presented the major influence on the glycan binding, favoring the approach of both Lys614 and Lys833 to the ligand by changes in the hydrophobic cleft, thereby altering its conformation. To date, the His716 imidazole group is thought to act as a base catalyst for the sulfuryl transfer, activating the glucosamine N-linked hydroxyl nucleophile assisted by lysine residues, while PAP exits the stabilized complex [13]. Moreover, His716 may play a role in stabilizing the transfer of the sulfuryl group [13], [16]–[18].

A serine residue close to the catalytic pocket conserved in all known STs binds to PAPS, shifting the enzyme conformation as to favor interaction of PAPS with the catalytic lysine residue [4], [19]. This Ser-Lys interaction removes the nitrogen side chain of the catalytic Lys from the bridging oxygen, preventing PAPS hydrolysis. Interestingly, the Lys614Ala mutant displays a hydrogen bond between PAPS 3′ Oγ and the Ser832 side-chain, thus implicating involvement of Lys614 in PAPS stabilization, which has previously been described in other sulfotransferases [19]. The His716Ala mutant displayed weaker docking energy for the PAPS/α-GlcN-(1→4)-GlcA complex when compared to the native enzyme, indicating a decreased molecular interaction between the ligand and acceptor.

**Figure 3:**
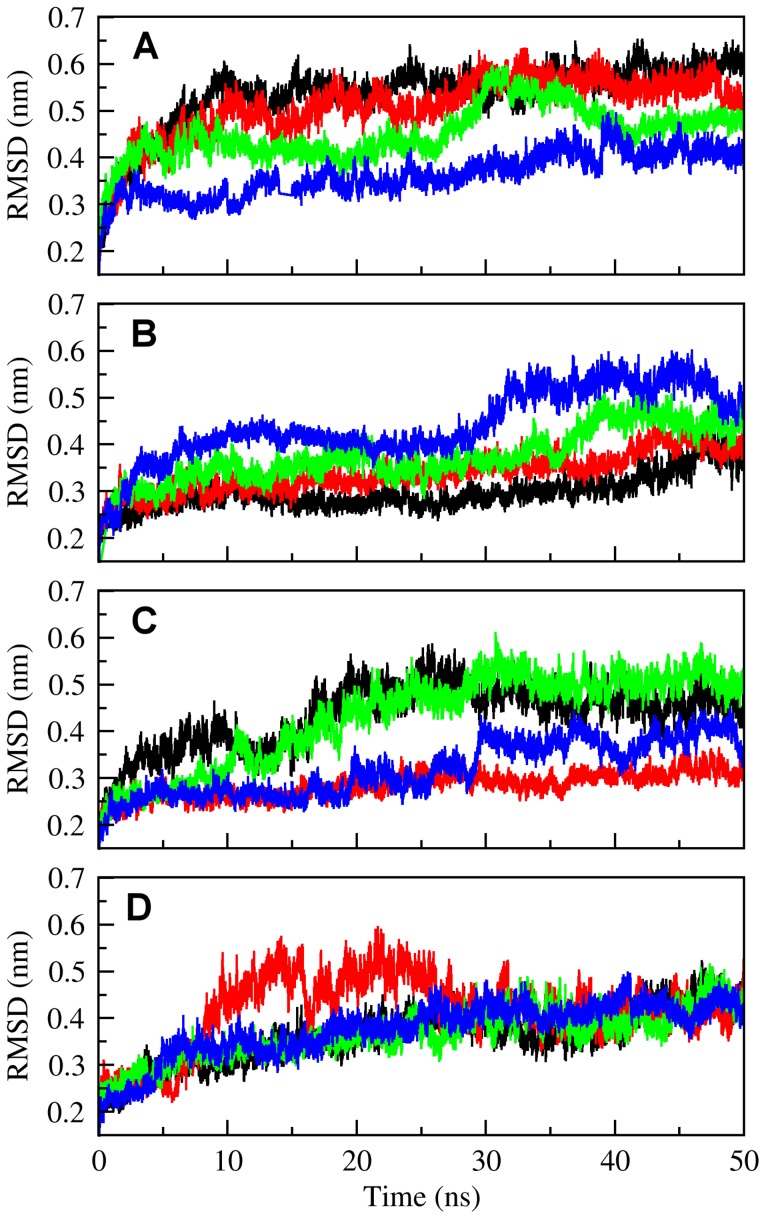
All-atom root-mean-square deviation (RMSD) of the protein, plotted against the 50 ns MD simulation time, for the systems containing (A) the NST alone and for the (B) NST/PAPS, (C) NST/PAPS/α-GlcN-(1→4)-GlcA and (D) NST/PAP/α-GlcNS-(1→4)-GlcA complexes. Black, NST-1; Green, Lys614Ala; Blue, His716Ala, Red, Lys833Ala.

*Molecular Dynamics Simulation* – To search for associations between local/global conformational changes and the substrate binding to the enzyme, MD simulations were performed for the complexes that resulted from docking analysis, as well as mutated, bonded and unbounded proteins. Accordingly, in order to examine conformational variations of the NST during simulations, the root-mean-square deviation (RMSD) of the Cα atomic positions with respect to the crystal structure were evaluated for the native protein and three mutants (Fig. 3). As a general feature, the obtained RMSD values achieved a plateau after the first 10 nanoseconds, with little conformational changes during their passage through plateaus. The analyses of the RMSD values of NST all-atom for the NST/PAPS complex, NST/disaccharide/PAPS complex and native enzyme alone showed that the NST/PAPS complex is relatively more stable (Fig. 3A and B), with lower RMSD fluctuations, compared to native enzyme, PAPS/α-GlcN-(1→4)-GlcA and PAP/α-GlcNS-(1→4)-GlcA complexes (Fig. 3C and D). The complex NST/PAP/α-GlcNS-(1→4)-GlcA (black) MD simulations presents a decrease in RMSD fluctuations over time due to the eventual stabilization of the substrate/enzyme complex which shifts to a stable orientation/conformation after an initial rearrangement. In order to acquire specific data on disaccharide positioning and fluctuations during the simulation, the RMSD for the disaccharide in relation to NST complexes were obtained based on the MD simulations. The RMSD of α-GlcN-(1→4)-GlcA atoms rose to 2.0 Å after 3 ns, presenting fluctuating peaks with this maximum amplitude during the entire simulation, indicating that an equilibrium state is not achieved for the non-sulfated moiety during the simulation in the presence of PAPS (Fig. S3). This fluctuation on RMSD is also observed using an octasaccharide as ligand (data not shown). Interestingly, the RMSD values for the mutant models, although increased, were more stable, reflecting the influence of these residues in the enzyme catalysis (Fig. 3C and D). Time-dependent secondary structure fluctuations were analyzed using the DSSP program [20], and most of the secondary structures (such as the β-sheet and α-helix) from the initial structure remained stable (Fig. S4a–d).

### Interaction Energy

**Figure 4:**
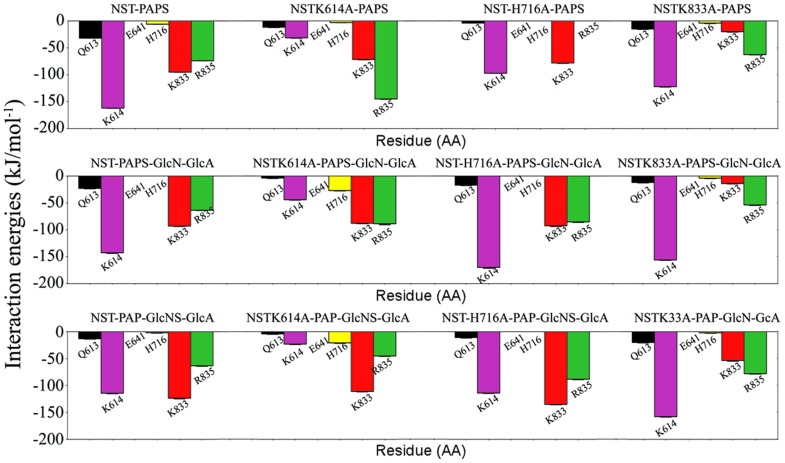
Per residue interaction energies between NST sidechain residues and sulfate in both PAPS and disaccharide models.

The contribution of specific amino acid residues for the interaction between NST and PAPS, as well as between NST/PAPS and disaccharides, was calculated using the program g_energy from GROMACS-4.5.1 package [21], and their respective average values, for the entire simulation time, are presented in Fig. 4. The interaction energy profile of NST/PAPS/α-GlcN-(1→4)-GlcA complex is always more intense than that of NST/PAP/α-GlcNS-(1→4)-GlcA complex, indicating stronger binding of the disaccharide to NST/PAPS compared to the binding to NST/PAP complex. The predicted binding energies (kJ.mol^−1^) may be translated into dissociation constants in the µM range, indicating strong binding. In order to evaluate the effect of distinct residues on ligand binding, we performed a per-residue calculation of the energetic influences of critical residues on the binding. Fig. 3 lists the average energy contributions of these key residues. Moreover, the electrostatic interaction between sulfate from ligands (PAPS or α-GlcNS-(1→4)-GlcA) and the positively charged residues Lys614 and Lys833 are the dominant contributions to the binding of these ligands. These results agree with our molecular docking data, where these residues were shown to act as anchors for the sulfate donor moiety from PAPS.

### Essential Dynamics (ED)

**Figure 5:**
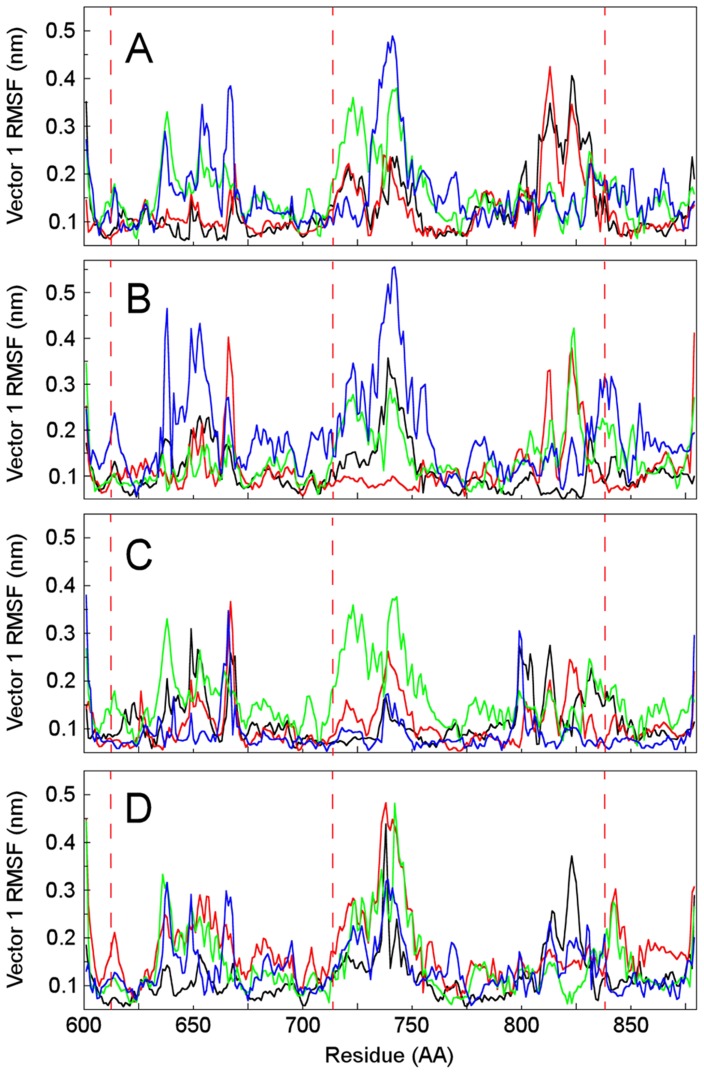
CαRMSF of the first eigenvector as a function of residue number. Black, NST; green, NSTLys614Ala; blue, NSTHis716Ala; red, NSTLys833Ala. A, N-sulfotransferase domain (NST) alone; B, NST-PAPS systems; C, NST-PAPS-GlcN-GlcA; D, NST-PAP-GlcNS-GlcA.

In order to investigate the motions of NST associated with the substrate binding, ED analyses were performed on the simulation trajectories containing: 1) NST/PAPS complexed to the unsulfated disaccharide (α-GlcN-(1→4)-GlcA), and 2) NST/PAP complexed to the sulfated disaccharide (α-GlcNS-(1→4)-GlcA). The differences in the dynamics of the active site observed in the complex with α-GlcN-(1→4)-GlcA and PAPS, considering the major residues responsible for binding, are reflected at the level of global flexibility. Analysis of residue-based RMSF (Root Mean Square Fluctuations) after projection along the main ED eigenvectors indicates that the dynamic motions of the NST/PAPS complex are distributed throughout the protein domain, with little fluctuation along the principal direction of motion (Fig. 5). The cosine contents with 0.5 periods for the projections of the eigenvector 1 are close to zero, indicating that complete sampling/equilibrium has been achieved (Table 2). In both uncomplexed and PAPS complexed NST, the mutation of Lys614 affects the motions of the 3′ PB loop that contains the Lys833 residue, whereas mutation of this last residue affects the motions of 5′ PSB, where Lys614 is located (Fig. 5A and B). The disaccharide binding also affects the motions of this vector, fluctuating along the principal direction of motion with a characteristic involvement of Lys614, Lys833 and His716 containing regions of increasing global flexibility at the active site during sulfate transfer, whereas in the conformational equilibrium of both 3′ PB (α6 helix) and 5′ PSB loop tends to be shifted toward more relaxed nonfunctional state.

**Table 2:** Cosine Content of the First Three Eigenvectors.

	PC1	PC2	PC3
NST	0.0152	0.0065	0.0008
NST614	0.0168	0.0109	0.0013
NST716	0.0074	0.0017	0.0003
NST833	0.0227	0.0087	0.0022
NST-PAPS	0.0099	0.0034	0.0017
NST614-PAPS	0.0087	0.0025	0.0014
NST716-PAPS	0.0051	0.0011	0.0002
NST833_PAPS	0.0092	0.0057	0.0021
NST-PAPS-GLC	0.0247	0.0103	0.0081
NST614-PAPS-GLC	0.0210	0.0087	0.0038
NST716-PAPS-GLC	0.0092	0.0015	0.0009
NST833-PAPS-GLC	0.0276	0.0121	0.0058
NST-PAPS-GLC	0.0180	0.0068	0.0022
NST614-PAPS-GLC	0.0093	0.0026	0.0013
NST716-PAPS-GLC	0.0119	0.0035	0.0019
NST833_PAPS-GLC	0.0143	0.0055	0.0022

### Changes in Molecular Motions upon PAPS PCA of Combined MD Trajectories

**Figure 6:**
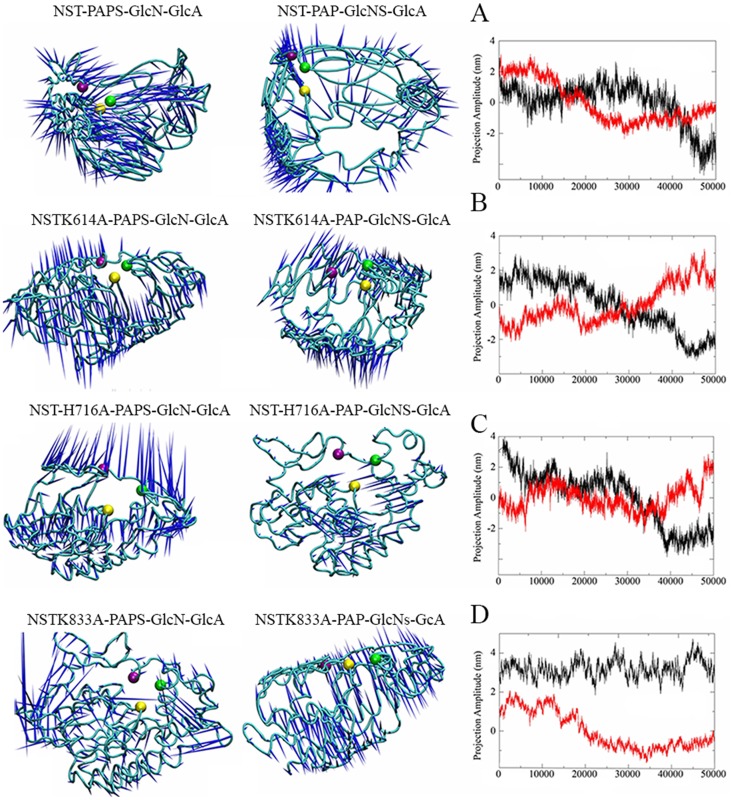
Effect of mutated residues in structural conformational changes. Computational dynamic analysis of NST is shown as cyan Cα trace in each model. Porcupine plots showing the direction and amplitude of conformational changes between PAPS/GlcN-GlcA and PAP/GlcNS-GlcA states represented by the first eigenvector of the principal mode Cα atoms calculated from the 50 ns simulation. The orientation of the blue cone indicates the direction of motion of the atom, and its length is proportional to the amplitude of the motion. Predicted binding residues are shown: yellow, Lys614; green, His716; and purple, Lys833. Right column: principal component analysis of combined MD trajectory of NST/PAPS/GlcN-GlcA and NST/PAP/GlcNS-GlcA and mutants. Projection of the MD trajectories on the first eigenvector of the covariance matrix of Cα atoms. Black, projections of the first 50 ns of the combined trajectory NST-PAPS-GlcN-GlcA; red, projections of the 50 of the combined trajectory NST-PAP-GlcNS-GlcA. N-sulfotransferase domain and Lys614, His716 and Lys833 are represented in figures A-D.

To extract functionally relevant, large-scale cooperative motions, we performed an ED analysis on the NST/PAPS/α-GlcN-(1→4)-GlcA and NST/PAP/α-GlcNS-(1→4)-GlcA trajectories. Eigenvalues rapidly decreased, whereas the first 2 eigenvectors contributed the most to the fluctuation (Fig. 6), accounting for the major percentage of the total fluctuations in the free form, PAPS ligated, and both NST-PAPS-α-GlcN-(1→4)-GlcA and NST-PAP-α-GlcNS-(1→4)-GlcA, respectively (data not shown). Projection of the original MD trajectories on the eigenvectors generated from ED analysis produces principal components, representing the directional motions on the course of the simulation. The cosine content of a principal component can be used as an indicator to determine whether the sampling of an MD simulation converges. We therefore calculated the cosine content of the first two principal components to determine if the convergences were obtained during the MD simulations (Table 2). The cosine content of the principal components was remarkably small for the free form and PAPS binding NST and mutants, indicating that the diffusive content of these eigenvectors was relatively low and thus reveal converged conformational transitions.

The projected MD trajectories for the NST/PAPS/α-GlcN-(1→4)-GlcA and NST/PAP/α-GlcNS-(1→4)-GlcA complexes along the first eigenvector also points to the relevance of the motions for glycan binding. Accordingly, it is possible to observe a clear separation between the motions of PAPS/α-GlcN-(1→4)-GlcA and PAP/α-GlcNS-(1→4)-GlcA along eigenvector 1 in mutant NST614A (Fig. 6B), suggesting that the correlated motions represented by this vector may reflect important conformational changes associated with ligand binding. We therefore used eigenvector 1 to filter the MD trajectories and isolate the intra subunit and inter subunit motions associated with this component). When bound to the disaccharide, the differences between the extreme structures were more evenly distributed over the whole protein (Fig. 6A). In the NST/PAPS/α-GlcN-(1→4)-GlcA simulations, a large directional motion is visible along the α6 that constitutes the opposing face of the glycan binding cleft, where His716 is located. This may be correlated to its first motion in deprotonating the acceptor. The NST/PAP/α-GlcNS-(1→4)-GlcA simulations, on the other hand, effectively reduced the largest directional motion of this region, which corroborates with the idea that the dynamic behavior in regions opposite the substrate-binding site could play a role in modulating the dynamics of the substrate-binding pockets [22], [23]. Combining the observations that the coil containing Lys833 has the largest movement in the PAPS/α-GlcN-(1→4)-GlcA and PAP/α-GlcNS-(1→4)-GlcA and its location at one of the openings of the α6 cleft, we speculate that this turn promotes Lys833 coordination with the bridge oxygen in this alternative binding site.

### Binding

Figure 5 shows the mean square displacements (RMSF) of the first eigenvector as a function of residue number. Several large conformational arrangements are observed in NST upon substrate binding, and regions showing relatively large shifts (CαRMSF >0.06 nm) comprise residues 610–621 (helix-1), 630–675 (helix 2 and 3), 710–732 (helix 6 and 7), 741–755 (helix 9), 810–848 (β-strand 1/2 and loop). Among these, the most significant conformational shifts (RMSF >0.3 nm) occur in the α-helix 6, 9 and the loop containing Lys833, which is unique to NST, when compared to other sulfotransferases. Inspection of the motions along eigenvector 1 reveals that the mutation of Lys614 increases the motion of the Lys833 loop, whereas mutation of Lys833 affects both α-helix 1 and α-helix 6, which constitute the open cleft substrate-binding site. Mutation of His716 also increases the motion of α-helix 1, which might correlate with its involvement in the stabilization of PAPS and the hydroxyl group deprotonation of the substrate and subsequent attack of the sulfur atom from PAPS. Upon PAPS binding, the structural changes originate mainly from the regions of residues from helix 6 and 7 in the native enzyme, indicating that the displacement of this segment is capable of mediating structural changes in the loop region 810–848 and thus in the accommodation of the incoming substrate.

### Changes in Molecular Motions upon Disaccharide Binding

The RMSD of simulations revealed that the open cleft forms of the protein (sweet hill, helix 6 and loop containing Lys833) exhibit a much larger conformational drift from the initial structure (up to 3.8 Å in the case of the NST His716Ala simulation). There are three large conformational drifts, visualized as peaks in all simulations, that show a large degree of fluctuation compared to the rest of the protein. This simulation shows that in the Lys833Ala mutant, the relative PAPS-binding domain motions decrease in comparison to the NST/PAPS simulation alone. On the other hand, an increase in the motion is observed for NSTLys614Ala and NSTLys716Ala mutants. The large-scale concerted motions of the unsulfated and sulfated disaccharide ensembles can be shown in the extremes of the porcupine representation (Fig. 6). The most relevant motions of the NST and its mutated models in different conformational forms, as described by eigenvector 1, are around the random coil containing Lys833 and the α-helix 6. In the presence of the ligand in the binding cleft, the subdomains would be expected to close as to readily accept a ligand. However, the closing motions of the enzyme appear to be highly affected in the Lys833Ala mutant.

### Water Involvement in Sulfate Transfer

**Figure 7:**
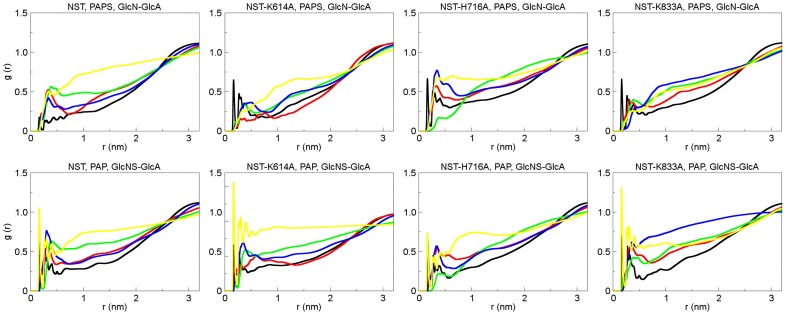
Radial distribution functions. g(r), centered on the side chain atoms of the residues involved in sulfate transfer to the oxygen atoms of modeled water of the eight complexes: Black, Sulfonate Oγ solvation; red, Lys614 Nγ solvation; green, His716 NHτ solvation, blue, Lys833 Nγ solvation; yellow, glycan NH2 solvation.

The RDFs (Radial Distribution Functions) for hydrogen bond related to residues analyzed of the four models: side-chain Nγ atom from Lys614, Lys833 and His716; sulfate from PAPS and sulfated disaccharide and NH_2_GlcN-GlcA to estimate the total number of water molecules in the pocket of the NST (Fig. 7).

Radial Distribution Functions (RDFs) RDFs describe the ratio between the local density of water molecules around a reference site rP and the average density ρ of water molecules in the solution, meaning the probability of finding the particle of type y in the spherical radius r around the particle of type x (RDFs, gxy(r)).

Two prominent maxima can be found in the sulfate, Lys614, Lys833, indicating that two solvation shells exist around those residues prior catalysis (Fig 7A). The sulfate oxygens give rise to an RDF with multiple peaks. The first peak around the PAPS shows that the first coordination shell of water around the sulfate group is within 0.2 nm, which corresponds to a position of one water molecule near one of the two sulfate-oxygens. The second and third peaks, which are at 0.32 and 0.36 nm, correspond to a situation where one water molecule is coordinated with both sulfate-oxygens. Similar values for the first peak are found for both Lys614 and Lys833. The first maximum becomes especially sharp for the NST/PAP/α-GlcNS-(1→4)-GlcA sulfate (Fig 7B) with a corresponding CN of 0.6 nm, suggesting that the first hydration shell is well established in the vicinity of the sulfate atom. Mutations at Lys614 and Lys833 residues influences the solvation of each other, possibly by destabilizing the water of the active site cavity (Figs 7B–D; F–H). This data suggests that water molecules are at close distance to sulfate group and may participate on bridging the sulfate and Lys.

## Discussion

A molecular docking and molecular dynamics approach was used to study in detail the sulfotransferase domain of human *N*-deacetylase *N*-sulfotransferase (NDST) and decipher the catalytic relevance of the boundary residues through the hydrophobic cleft, as well as the role of critical amino acid residues for ligand binding.

The obtained model for the substrate recognition by *N*-deacetylase *N*-sulfotransferase 1 reveals residues that interact with the acceptor substrate. The subsequent mutation of possible catalytic residues provided structural evidence that these residues are involved in substrate binding and/or catalysis. Although NST exhibits some unique structural features, such as the presence of the second potential catalytic base Lys833, the underlying mechanism of the reaction catalyzed by NST appears to be similar to that of estrogen sulfotransferases (ESTs) and other *O*-sulfotransferases (OSTs), in which the conserved catalytic residues act in concert in order to advance the reaction. Our present substrate-binding model should serve as a promising template for the general structure and function of heparan sulfate/heparin *N*-and *O*-sulfotransferases.

In the current study, strictly conserved regions of NST (5′PSB and 3′PB), involved in the sulfate transfer from PAPS (universal sulfate donor) to a glycan residue, were described. These results agree with previous biochemical findings [4], [18], [24], where a conserved Lys may induce a charge build up around the sulfate group. In addition to catalytic active site residues reported previously, were confirmed the potential functions for additional Lys833 on both sulfate donor and glycan acceptor, reinforcing previous empirical investigations of the roles of these residues in the active site formation [18], [25], [26]. A favorable water-interaction after mutation of catalytic residues might be induced by some degree of electronic polarization in nearby water molecules. From the obtained data, it may also be evidenced that the favorable interactions between enzyme and saccharide are not maintained in either one of the three studied mutants.

To our knowledge, this is the first computational report on the glycosaminoglycan N-sulfation process using PAPS, offering critical information on the ways in which the interaction between the N-sulfotransferase domain and the sugar moiety occurs in both structural and dynamical behaviors. In addition, a set of simulations using PAP and the sulfated disaccharide was performed in order to evaluate the end points of the reaction pathway. PAP is known to function as a strong inhibitor of sulfotransferases [27], [28], reflecting in a global decrease of the interaction energies within the enzyme and disaccharide.

Unlike the syntheses of nucleic acids and proteins, which are template-driven processes, the biosynthesis of glycosaminoglycans involves multifactorial mechanism which leads to the immense variability noted in these classes of sugars. The interaction between biosynthetic enzymes, as well as, the affinity of these enzymes/enzyme complexes to the sugar chain plays a major role in the final glycosaminoglycan structure. Therefore, studies which unveil substrate and enzyme inhibition patterns directly impact the understanding of regulating the glycosaminoglycan fine structure. Our results shed light on amino acids within and around the NST active site which directly modulate the affinity of the enzyme to the sugar chain.

The ability to study intermediate states of the enzymatic reaction provides insights into the precise role each amino-acid plays, and thus information could be used to improve chemoenzymatic production of heparin and HS.

## Materials and Methods

### Software and Nomenclature

All saccharide and PAPS topologies were generated using the PRODRG server [29]. Posterior manipulation of the structures was performed using MOLDEN [30], to draw ligand structures and handle ab initio-derived files, VMD [31], for visualization of the trajectories, and PyMOL, to generate the NST mutants [32]. Molecular dynamics (MD), including both calculations and analyses, was performed using GROMACS simulation suite, version 4.5.1 and GROMOS96 43a1 force field [33]. The relative orientation of a pair of contiguous carbohydrate residues is described by two or three torsional angles at the glycosidic linkage. For an (1→4) linkage, the Φ and Ψ were defined as shown in the equations 1 and 2:
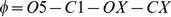
(1)

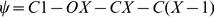
(2)


### Atomic Charge Calculation

The calculation of atomic charges suitable for the PAPS MD simulations was performed as previously described for other sulfated compounds [34], [35]. Briefly, the PAPS was submitted to full geometry optimization at the HF/3-21G level using GAMESS [36]. Subsequently, the minimal energy conformations were submitted to single-point calculations at the HF/6-31G** level in order to obtain the Löwdin derived charges [37] (Fig. S5). Hessian matrix analyses were employed to unequivocally characterize the conformations thus obtained as true minima potential energy surfaces.

### Disaccharide Topology Construction and Energy Contour Plot Calculation

To obtain a conformational description of the glycosidic linkages associated with the studied saccharides, the composing fragments were constructed using MOLDEN software [30]. These structures were then submitted to the PRODRG server [29], and the initial geometries and crude topologies retrieved. Such disaccharide topologies were further modified to include some refinements: (1) improper dihedrals, employed to preserve the conformational state of the hexopyranose rings in ^4^C_1_ (d-GlcN, d-GlcA), ^1^C_4_ (l-IdoA) forms; (2) proper dihedrals, as described in GROMOS96 43a1 force field for glucose, in order to support stable simulations [38], and (3) Löwdin HF/6-31G** derived atomic charges, which were either obtained from previous works [34], [35], or calculated (Fig. S6). The conformational description of glycosidic linkages was performed by varying φ and ψ angles, formed by two consecutive monosaccharide residues, from −180 to 150 degrees with a 30 degree step, in a total of 144 conformers for each linkage, as previously described [39], [40]. A constant force was employed restricting only φ and ψ proper dihedrals during energy minimization in each of the afore-mentioned values, allowing the search of the conformational space associated with the linkage. Thereafter, using minimized output conformations, a series of MD simulations were performed for 20 picoseconds (ps) at 10 K, with an integration step of 0.5 femtoseconds (fs), to further reinforce the search for minimum-energy states. The relative stabilities of each conformation, obtained from the 10 K MD last frame, were used to construct relaxed energy contour plots (Fig. S7) describing the conformation of each glycosidic linkage.

### Docking Procedures

AutoDock4.2 was used as grid-based docking procedure [41]. Although heparan sulfate docking validation against crystal structures such as IL9 and have been performed elsewhere [42], we performed a docking using the 1.8 Å resolution structure of 3-O-sulfotransferase bound to a heptasaccharide substrate using Autdock (PDBiD 1T8T). The obtained reference RMSD was 0.49 for the lowest scoring energy population (−12 Kcal/mol –table S1). The crystal structure of the sulfotransferase domain of human heparan sulfate *N*-deacetylase/N-sulfotransferase 1 bound to PAP obtained from Brookhaven Protein Data Bank (PDB ID code: 1NST) [25] was used in the docking experiments. Missing side chain atoms and the Kollman united atom partial charges to the PAPS molecule were included [43], [44]. Concerning carbohydrate structures, the Löwdin atomic charges, as previously calculated for sulfated saccharides [39], [40], were employed and all torsion angles were considered flexible. The grid maps, calculated using AutoGrid, were chosen to be large enough to include the active site, as well as a significant portion of the surrounding surface. The dimensions of the grids were thus 50 Å × 50 Å × 40 Å, with 0.3 Å spacing between the grid points. Docking of the disaccharide to 1NST was carried out using the empirical free energy function and the Lamarckian genetic algorithm, applying a standard protocol with an initial population of 500 randomly placed individuals, a maximum number of 2.5×10^8^ energy evaluations, a 0.02 mutation rate, a 0.80 crossover rate, and an elitism value of 1, where the average of the worst energy was calculated over a window of the previous 10 generations. One hundred independent docking runs were carried out for the disaccharide. Results were clustered according to the 0.5 Å root-mean-square deviation (RMSD) criteria.

### MD Simulations

The sixteen molecular systems to undergo MD, which presented ∼35.000 atom each, were built comprising the NST domain of NDST, mutants for Lys614, His716 and Lys833 residues and different complexation states. Namely, (1) unbound, wild ST domain, (2) unbound, Lys614Ala mutated ST domain, (3) unbound, His716Ala mutated ST domain, (4) unbound, Lys833Ala mutated ST domain, (5)PAPS complexed to wild ST domain, (6) PAPS complexed to Lys614Ala mutated ST domain, (7) PAPS complexed to His716Ala mutated ST domain, (8) PAPS complexed to Lys833Ala mutated ST domain, (9) unsulfated disaccharide/PAPS complexed to wild ST domain, (10) unsulfated disaccharide/PAPS complexed to Lys614Ala mutated ST domain, (11) unsulfated disaccharide/PAPS complexed to His716Ala mutated ST domain, (12) unsulfated disaccharide/PAPS complexed to Lys833Ala mutated ST domain, (13) sulfated disaccharide/PAP complexed to wild ST domain, (14) sulfated disaccharide/PAP complexed to Lys614Ala mutated ST domain, (15) sulfated disaccharide/PAP complexed to His716Ala mutated ST domain, and (16) sulfated disaccharide/PAP complexed to Lys833Ala mutated ST domain. Such systems, as well as the minimum-energy conformations obtained from the energy maps for the disaccharides, were solvated in rectangular boxes using periodic boundary conditions and SPC water model [45]. Counter ions (Na^+^, Cl^−^) were added to neutralize the system, whenever needed. The employed MD protocol was based on previous studies [34], [35], [46]. The Lincs method [47] was applied to constrain covalent bond lengths, allowing an integration step of 2 fs after an initial energy minimization using Steepest Descents algorithm. Electrostatic interactions were calculated using Particle Mesh Ewald method [48]. Temperature and pressure were kept constant by coupling protein, carbohydrates, PAPS, ions and solvent to external temperature and pressure baths with coupling constants of τ  = 0.1 and 0.5 ps [49], respectively. The dielectric constant was treated as ε  = 1. The systems were heated slowly from 50 to 310 K, in steps of 5 ps, each one increasing the reference temperature by 50 K. After this heating, all simulations were further extended to 50ns under a constant temperature of 310K. Hydrogen bonds were defined when the donor-acceptor heavy atom distance was 0.35 nm and the acceptor atom–donor hydrogen angle was 30 degrees.

### Essential Dynamics (ED)

ED analysis was performed in order to filter the large concerted motions of NST during substrate binding. This method is based on the diagonalization of a covariance matrix of atomic fluctuations, resulting in eigenvectors that indicate directions in a 3N-dimensional (N = number of atoms used for constructing the covariance matrix) configurational space. The eigenvalues represent the amplitude of the eigenvectors along the multidimensional space, and the displacement of atoms along each eigenvector shows the concerted motions of proteins in each direction. The resulting essential modes describe the mean-square fluctuation (MSF) of atoms in collective motions involving many atoms simultaneously, which can be used to discriminate dynamic behaviors between different simulations and mutants. The eigenvectors can then be ranked by decreasing eigenvalue, with the first and second eigenvector representing the largest contribution in the total fluctuation of the system, and its relative structures transformed back into Cartesian coordinates. The extreme projections along the eigenvector can then be interpolated. ED was carried out using the program g_covar from GROMACS-4.5.1 package [21]. The covariance matrix of positional fluctuation was computed for the 50 ns of each simulation for the Cα-atoms of residues 601–879 from NST domain. The overlap of the different covariance matrices was computed by pair wise alignment between all simulations with the program g_anaeig.

## Supporting Information

Figure S1 **Atom labels for both PAPS (A) and disaccharide (B).**
(TIF)

Figure S2 **Two-dimensional plots of the catalytic domain displaying PAPS, PAP and disaccharide interacting amino acids and bridging water molecules with details of hydrogen bond distances.** (A) NST/PAPS, (B) NST/PAPS/α-GlcN-(1→4)-GlcA and (C) NST/PAP/α-GlcNS-(1→4)-GlcA complexes. Light brown: interacting amino acids; Purple; PAPS; Orange; disaccharide.
(TIF)

Figure S3 **RMSD of α-GlcN-(1**→**4)-GlcA atoms during the course of simulation.** (A) NST/PAPS/α-GlcN-(1→4)-GlcA and (B) NST/PAP/α-GlcNS-(1→4)-GlcA complexes. Black, NST-1; Green, Lys614Ala; Blue, His716Ala, Red, Lys833Ala.
(TIF)

Figure S4 **Time-dependent secondary structure fluctuations were analyzed using the DSSP program.** (A) NST/PAPS, (B) NST/PAPS/α-GlcN-(1→4)-GlcA and (C) NST/PAP/α-GlcNS-(1→4)-GlcA.
(TIF)

Figure S5 Löwdin HF/6-31G** derived atomic charges calculated for both PAPS (A) and PAP(B) were used in both docking and molecular dynamics calculations.
(TIF)

Figure S6 **Relaxed energy contour plots describing the conformation of each glycosidic linkage showing the relative stabilities of each conformation, obtained from the 10 K MD last frame.** (A) α-GlcNAc-(1→4)-GlcA; (B) α-GlcNAc-(1→4)-IdoA; (C) α-GlcNS-(1→4)-GlcA; (D) α-GlcNS-(1→4)-IdoA
(TIF)

Figure S7 **Projection of trajectory onto the plane of first four eigenvectors.** Black; NST/PAPS/α-GlcN-(1→4)-GlcA and red, NST/PAP/α-GlcNS-(1→4)-GlcA.
(TIF)

Table S1 **Validation docking for 3-OST -3(PDBiD 1T8T) with heptasaccharide as obtained by Autodock 4.2 (Energy unit: Kcal/Mol).**
(DOCX)
