# Supplementary figures and images for: Insights into the N-Sulfation Mechanism: Molecular Dynamics Simulations of the N-Sulfotransferase Domain of Ndst1 and Mutants
Source: PLoS One. 2013 Aug 5;8(8):e70880. doi: 10.1371/journal.pone.0070880 (PMC3733922; doi:10.1371/journal.pone.0070880)

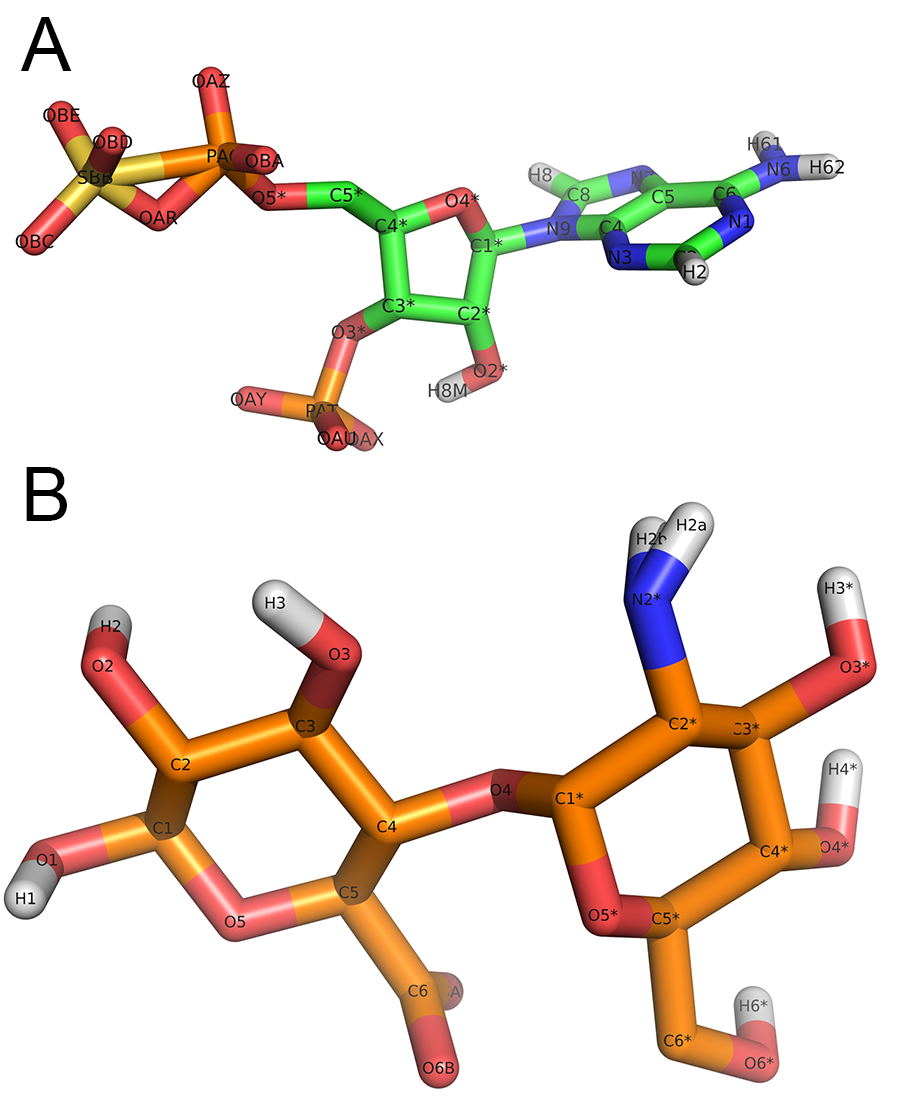

Supplement: Figure S1 — Atom labels for both PAPS (A) and disaccharide (B). (TIF) [file pone.0070880.s001.tif]

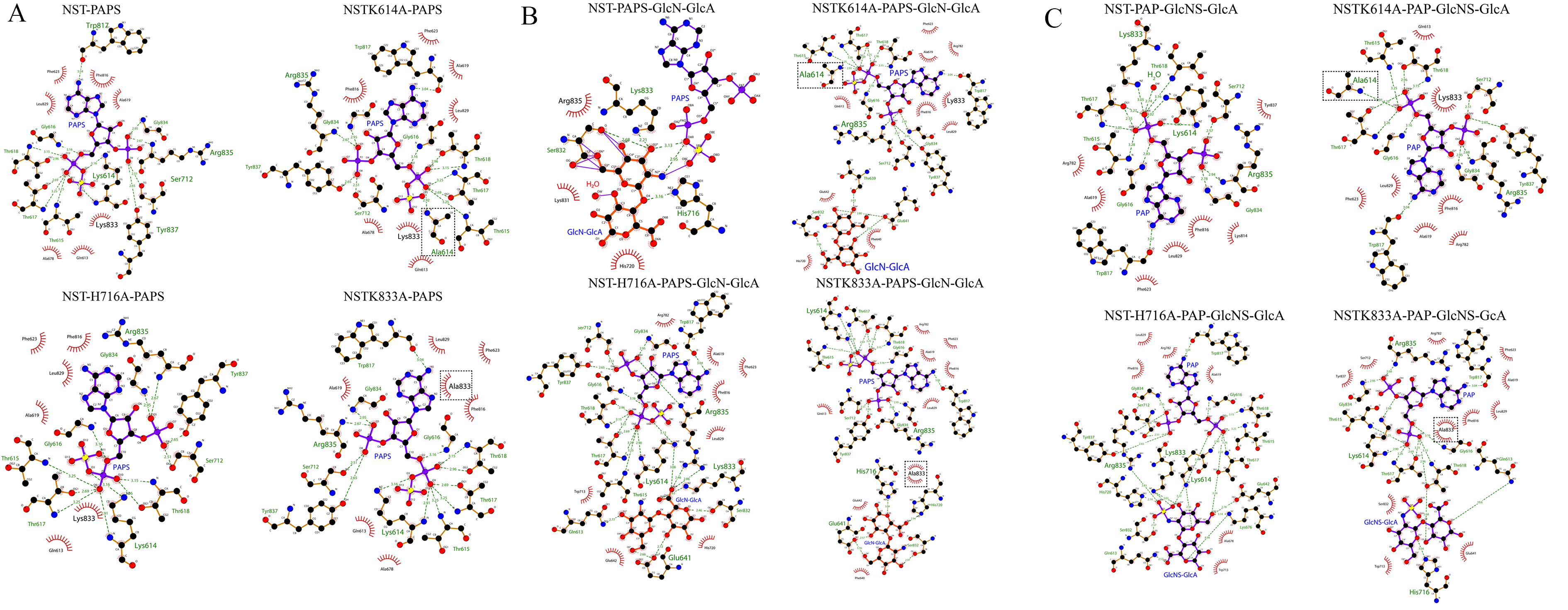

Supplement: Figure S2 — Two-dimensional plots of the catalytic domain displaying PAPS, PAP and disaccharide interacting amino acids and bridging water molecules with details of hydrogen bond distances. (A) NST/PAPS, (B) NST/PAPS/α-GlcN-(1→4)-GlcA and (C) NST/PAP/α-GlcNS-(1→4)-GlcA complexes. Light brown: interacting amino acids; Purple; PAPS; Orange; disaccharide. (TIF) [file pone.0070880.s002.tif]

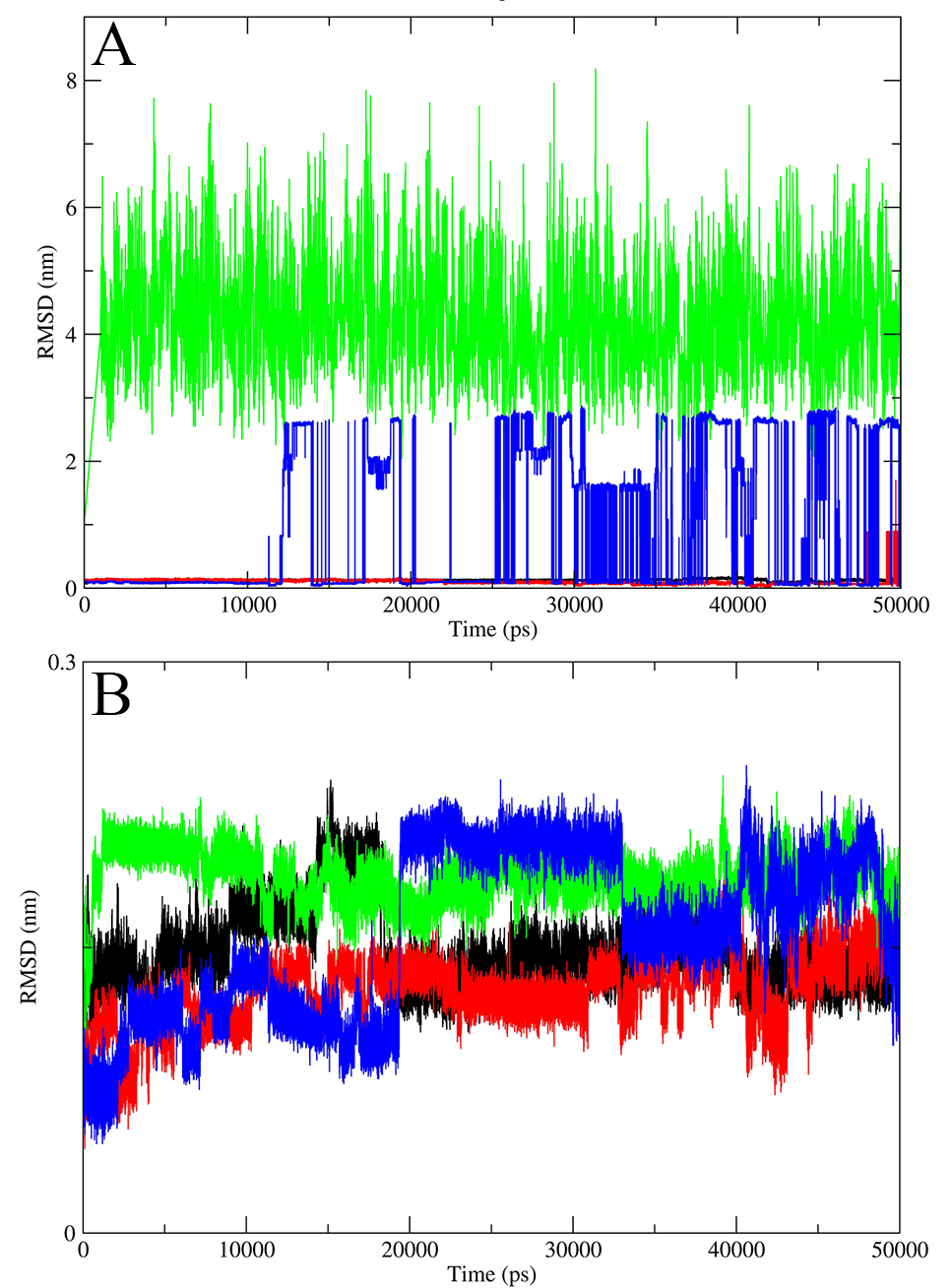

Supplement: Figure S3 — RMSD of α-GlcN-(1→4)-GlcA atoms during the course of simulation. (A) NST/PAPS/α-GlcN-(1→4)-GlcA and (B) NST/PAP/α-GlcNS-(1→4)-GlcA complexes. Black, NST-1; Green, Lys614Ala; Blue, His716Ala, Red, Lys833Ala. (TIF) [file pone.0070880.s003.tif]

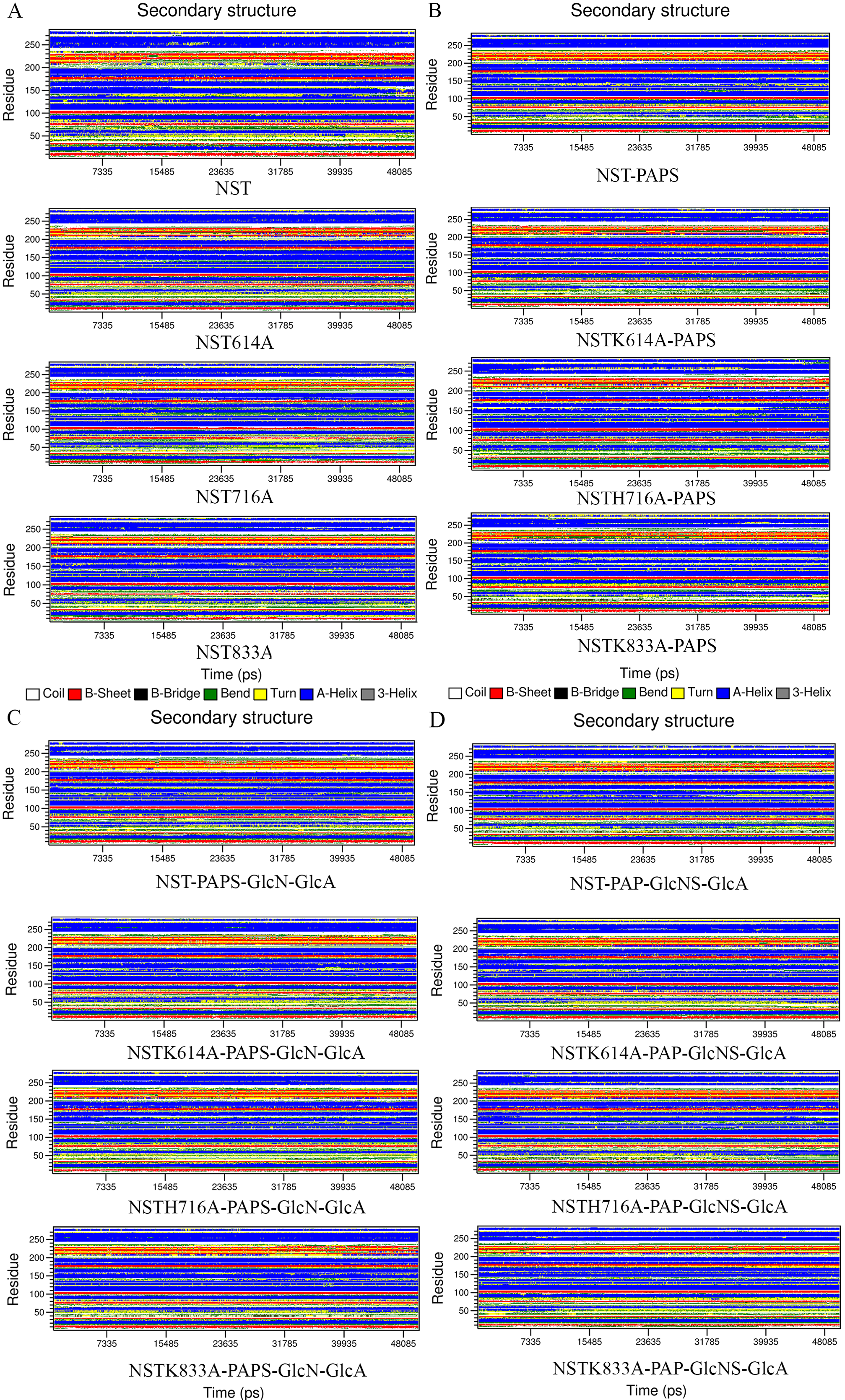

Supplement: Figure S4 — Time-dependent secondary structure fluctuations were analyzed using the DSSP program. (A) NST/PAPS, (B) NST/PAPS/α-GlcN-(1→4)-GlcA and (C) NST/PAP/α-GlcNS-(1→4)-GlcA. (TIF) [file pone.0070880.s004.tif]

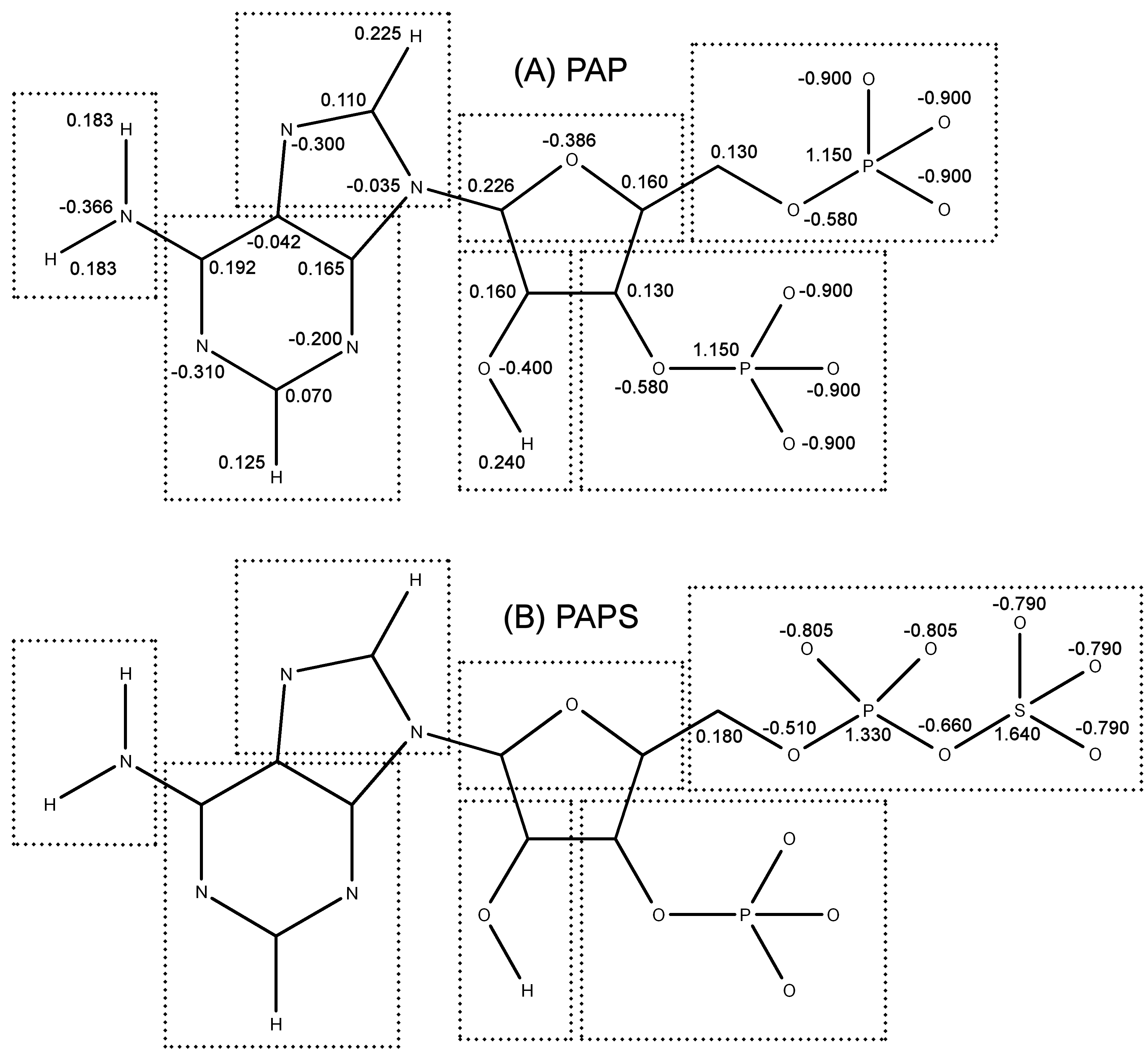

Supplement: Figure S5 — Löwdin HF/6-31G** derived atomic charges calculated for both PAPS (A) and PAP(B) were used in both docking and molecular dynamics calculations. (TIF) [file pone.0070880.s005.tif]

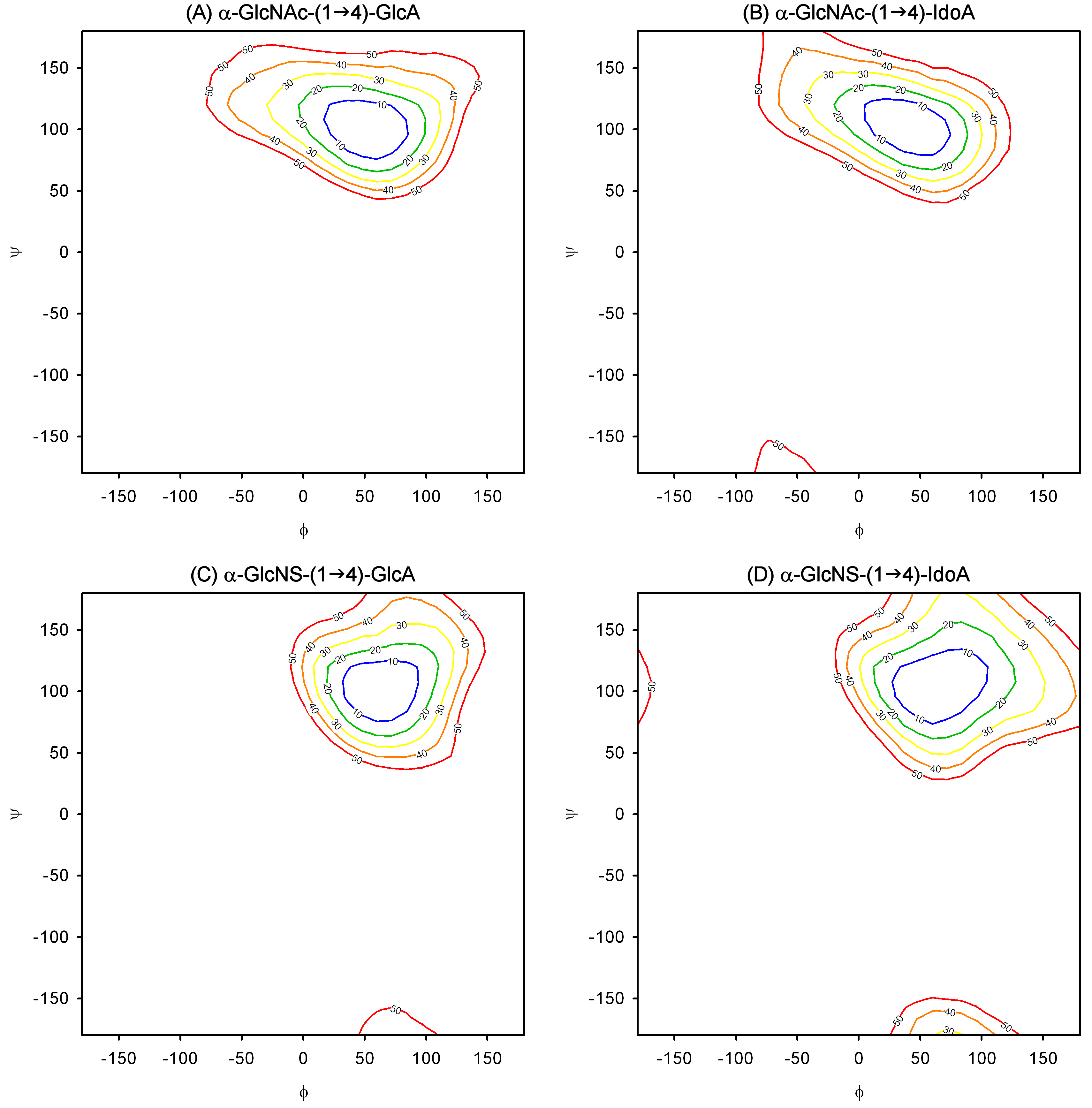

Supplement: Figure S6 — Relaxed energy contour plots describing the conformation of each glycosidic linkage showing the relative stabilities of each conformation, obtained from the 10 K MD last frame. (A) α-GlcNAc-(1→4)-GlcA; (B) α-GlcNAc-(1→4)-IdoA; (C) α-GlcNS-(1→4)-GlcA; (D) α-GlcNS-(1→4)-IdoA (TIF) [file pone.0070880.s006.tif]

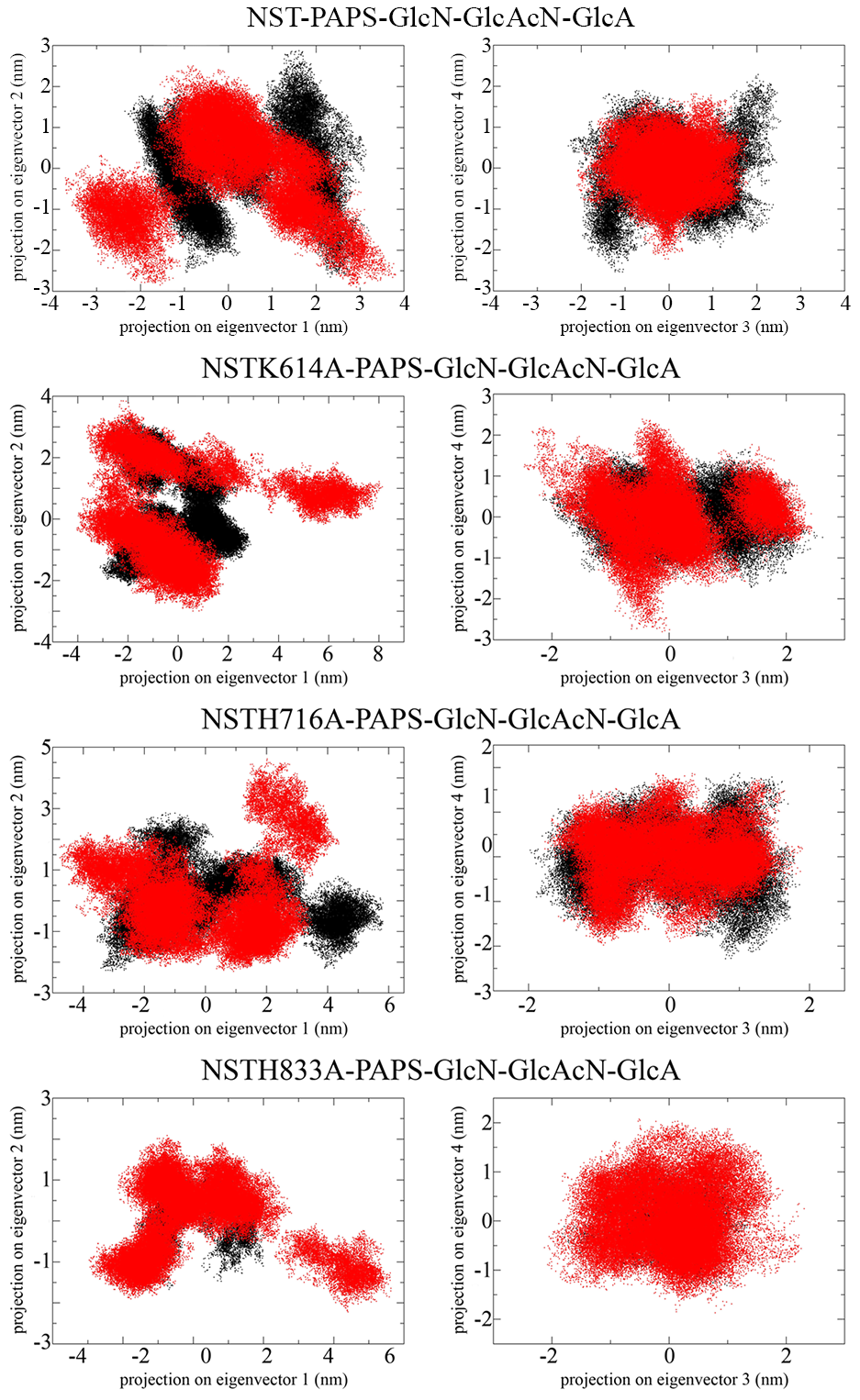

Supplement: Figure S7 — Projection of trajectory onto the plane of first four eigenvectors. Black; NST/PAPS/α-GlcN-(1→4)-GlcA and red, NST/PAP/α-GlcNS-(1→4)-GlcA. (TIF) [file pone.0070880.s007.tif]
