# Supplementary material for: Insights into the N-Sulfation Mechanism: Molecular Dynamics Simulations of the N-Sulfotransferase Domain of Ndst1 and Mutants
Source: PLoS One. 2013 Aug 5;8(8):e70880. doi: 10.1371/journal.pone.0070880 (PMC3733922; doi:10.1371/journal.pone.0070880)
Supplement: Table S1 — Validation docking for 3-OST -3(PDBiD 1T8T) with heptasaccharide as obtained by Autodock 4.2 (Energy unit: Kcal/Mol). (DOCX) [file pone.0070880.s008.docx]

| Clustering Histogram | | | | |
| --- | --- | --- | --- | --- |
| Lowest Binding Energy | Run | Mean Binding Energy | Num in Clus | Reference RMSD |
| -12.42 | 47 | -12.10 | 321 | 0.49 |
| -10.94 | 91 | -10.21 | 122 | 1.29 |
| -10.51 | 63 | -9.78 | 34 | 1.37 |
| -9.14 | 22 | -7.20 | 7 | 1.08 |
| -8.59 | 64 | -7.19 | 2 | 1.00 |
| -8.15 | 4 | -6.99 | 2 | 1.36 |
| -7.78 | 28 | -6.45 | 1 | 1.74 |
| -7.55 | 32 | -7.35 | 4 | 1.24 |
| -7.25 | 88 | -6.82 | 1 | 1.20 |
| -7.22 | 37 | -6.94 | 1 | 1.26 |
| -6.80 | 7 | -5.54 | 1 | 1.78 |
| -6.68 | 40 | -6.68 | 1 | 1.76 |
| -6.30 | 69 | -6.30 | 1 | 1.71 |
| -5.94 | 89 | -5.72 | 1 | 1.30 |
| -5.45 | 74 | -5.19 | 1 | 1.60 |

Supplemental Table I. Validation docking for 3-OST -3(PDBiD 1T8T) with heptasaccharide as obtained by Autodock 4.2 (Energy unit: Kcal/Mol).
